# Cost-effectiveness of Novel System of Mosquito Surveillance and Control, Brazil

**DOI:** 10.3201/eid1904.120117

**Published:** 2013-04

**Authors:** Kim M. Pepin, Cecilia Marques-Toledo, Luciano Scherer, Maira M. Morais, Brett Ellis, Alvaro E. Eiras

**Affiliations:** National Institutes of Health, Bethesda, Maryland, USA (K.M. Pepin);; Colorado State University, Fort Collins, Colorado, USA (K.M. Pepin);; Ecovec SA, Belo Horizonte, Brazil (C. Marques-Toledo, L. Scherer);; Universidade Federal de Minas Gerais, Belo Horizonte (M.M. Morais, A.E. Eiras);; Duke–National University of Singapore Graduate Medical School, Singapore (B. Ellis)

**Keywords:** dengue, dengue virus, viruses, mosquito surveillance, mosquito control, cost-effectiveness, Aedes aegypti, Brazil

## Abstract

Of all countries in the Western Hemisphere, Brazil has the highest economic losses caused by dengue fever. We evaluated the cost-effectiveness of a novel system of vector surveillance and control, Monitoramento Inteligente da Dengue (Intelligent Dengue Monitoring System [MID]), which was implemented in 21 cities in Minas Gerais, Brazil. Traps for adult female mosquitoes were spaced at 300-m intervals throughout each city. In cities that used MID, vector control was conducted specifically at high-risk sites (indicated through daily updates by MID). In control cities, vector control proceeded according to guidelines of the Brazilian government. We estimated that MID prevented 27,191 cases of dengue fever and saved an average of $227 (median $58) per case prevented, which saved approximately $364,517 in direct costs (health care and vector control) and $7,138,940 in lost wages (societal effect) annually. MID was more effective in cities with stronger economies and more cost-effective in cities with higher levels of mosquito infestation.

Dengue viruses cause ≈50 million infections annually worldwide, and ≈1% of these infections require hospitalization because of dengue hemorrhagic fever (*1*). Brazil accounts for ≈75% of all dengue cases in the Western Hemisphere (*2*), and during 2000–2005, Brazil reported more cases than any other country in the world (*3*). Since the reemergence of dengue in Brazil in 1982, there has been an epidemiologic shift to hyperendemicity (*4*,*5*) and more severe disease (*5*,*6*). Moreover, of all countries in the Western Hemisphere, Brazil has the highest economic losses caused by dengue ($1.35 billion) annually for direct medical and nonmedical costs and indirect costs from loss of work (*7*). This high economic cost of the disease occurs even after Brazil spent $1 billion annually on the dengue vector control program. Cost-effective methods of vector control are needed to decrease the huge economic effects of this disease in Brazil.

The most accurate method of assessing dengue risk by vector surveillance is one that specifically counts dengue vectors that are actively in search of a blood meal: adult female *Aedes aegypti* and occasionally *Ae. albopictus* mosquitoes. Ttraditional methods of vector monitoring in Brazil, which include surveys of larvae and pupae (*8*,*9*) and capture of adult mosquitoes by aspiration (*10*), are less specific and labor-intensive. Surveys of larvae target both vector sexes and can only predict the number of mosquitoes that will survive to adulthood, rather than directly measure adults. Capturing adults by aspiration does not specifically target female mosquitoes, is labor-intensive, and requires access to premises.

Fixed-position traps designed to capture gravid mosquitoes (e.g., MosquiTRAPs) (Ecovec SA, Belo Horizonte, Brazil) have been developed to reduce personnel costs and directly measure adult female mosquito abundance in Brazil (*11*,*12*). MosquiTRAPs have been implemented in the form of a large-scale mosquito surveillance system, Monitoramento Inteligente da Dengue (Intelligent Dengue Monitoring System [MID]; Ecovec SA), which is used to count mosquitoes in real time. MID involves weekly monitoring of MosquiTRAP (placed in a 300 m × 300 m grid format) counts and trapped-mosquito infection status with automated database updating (in situ mosquito data entry by cell phones directly to a Web-based database). The mosquito data are managed by a spin-off company (Ecovec SA), which provides daily updates to control personnel so they can specifically target highly infested areas. Preliminary results from 3 cities (Tres Lagoas in Mato Grosso do Sul State, and Presidente Epitacio and Bastos in Sao Paulo State) during 1 season of MID implementation showed that this system is effective in decreasing dengue cases (*13*). However, an estimate of cost-effectiveness for more cities over a longer period is needed for deciding whether MID should be maintained.

We evaluated the cost-effectiveness of supplementing vector control methods with MID in 21 cities in Minas Gerais State, Brazil, after use during 2 dengue seasons. We also identified factors that affected efficacy and cost-effectiveness of MID. We reported direct savings for health care costs and vector control activities separately from indirect savings for lost wages so that results are relevant to public health budgets and societal concerns.

## Methods

### Case Data

Monthly dengue cases during January 2007–June 2011 were obtained from each municipality in Minas Gerais, Brazil, by using Sinan Net (Information System for Notification of Grievances), a publicly available database of the Health Ministry of Brazil. Dengue cases were expressed as incidence per 100,000 inhabitants on the basis of the Brazilian Institute of Geography and Statistics (Rio de Janeiro, Brazil) 2010 population census.

### MID Mosquito Surveillance System

**Figure 1:**
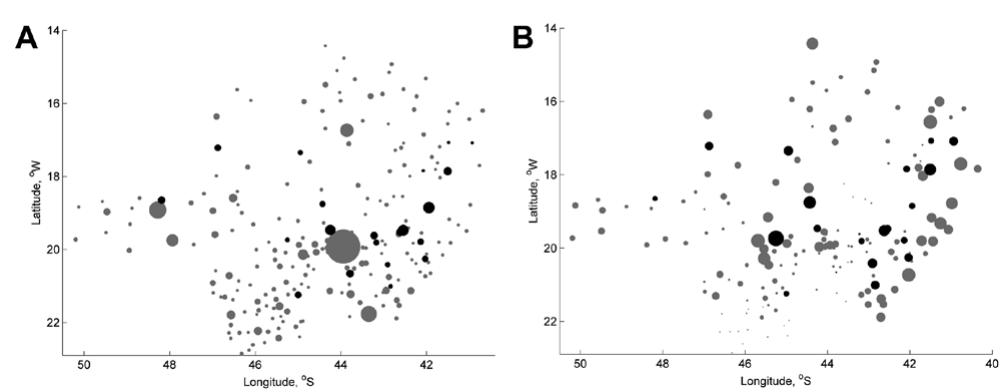
Spatial distribution of 21 cities tested with Monitoramento Inteligente da Dengue (Intelligent Dengue Monitoring System [MID]), Minas Gerais, Brazil, 2009–2011. A). Size of city centroids (n = 218) (circles) is proportional to population size. B) Size of city centroids (n = 147) (circles) is proportional to total dengue fever incidence during 2007–2011. Gray circles indicate cities that never implemented MID, and black circles indicate cities that implemented MID during mid-2009–June 2011. Areas of higher and lower total incidence are positively clustered with each other (Moran’s I, p<0.0001). Cities that implemented MID and those that had not implemented MID are distributed throughout areas of high and low incidence. Only cities with populations >15,000 are shown. Incidence data were not available for all cities.

MID was implemented in 21 cities in Minas Gerais during April 2009–June 2011. These cities are dispersed throughout the state in areas that included a range of population sizes and incidences (Figure 1). Cities that had the highest dengue incidence in the state were chosen by the Minas Gerais State Department of Health to receive MID. These cities were Aguas Formosas, Araguari, Bom Despacho, Caratinga, Conselheiro Lafaiete, Coronel Fabriciano, Curvelo, Governador Valadares, Ipatinga, Itabira, Joao Monlevade, Lavras, Malacacheta, Manhuaçu, Padre Paraiso, Paracatu, Pirapora, Ponte Nova, Sete Lagoas, Teofilo Otoni, and Visconde do Rio Branco. The only difference in vector-control activities between cities that used MID and those that did not use MID was that vector control in MID cities targeted sites that MID identified as highly infested with gravid adult mosquitoes. Details of the structure and function of MID and control efforts are shown in Technical Appendix 1.

### Data Analysis

**Figure 2:**
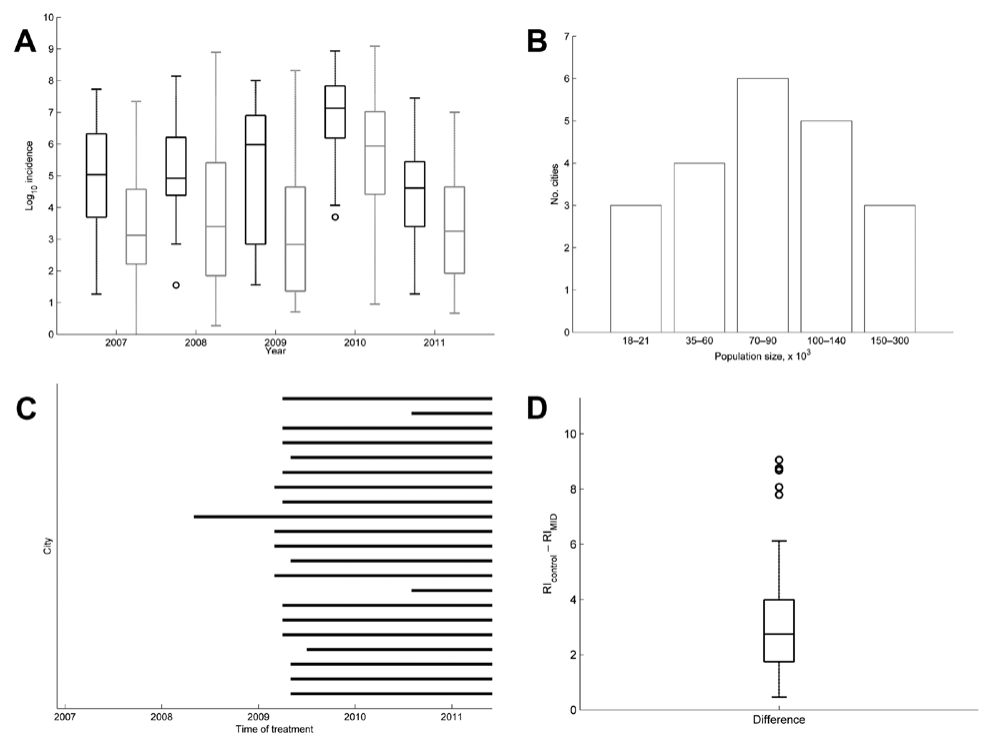
Changes in incidence of dengue fever in 21 cities that implemented Monitoramento Inteligente da Dengue (Intelligent Dengue Monitoring System [MID]), Minas Gerais, Brazil, mid-January 2007–June 2011. A) Annual incidence in 21 cities that implemented MID (bars outlined in black) and 147 cities that had not implemented MID (bars outlined in gray). Horizontal lines in boxplots indicate medians of 1,000 medians. Whiskers indicate ± 2.7 SD. Circles indicate points that fall outside ± 2.7 SD. B) Distribution of population sizes in cities that implemented MID. C) Time that MID was implemented in each city. D) Median relative increase (RI) in incidence for cities that implemented MID versus cities that had not implemented MID. RI was calculated as the sum of monthly incidence after MID was implemented divided by the sum of monthly incidence before MID was implemented for the same number of months. For cities that implemented MID, the median is a single value for the 21 cities. For cities that had not implemented MID, 21 cities with the same distribution of population sizes as MID cities were selected at random 1,000 times and their median relative differences during the same set of time frames were calculated. Horizontal line in the boxplot indicates median of 1,000 medians. Whiskers indicate ± 2.7 SD. Circles indicate points that fall outside ± 2.7 SD.

Dengue incidence was strongly seasonal, and outbreak probability varied substantially between cities (Figure 2, panel A), which did not follow any common statistical probability distribution in the exponential family. Thus, we adopted a nonparametric approach to data analysis. On the basis of potential differences in dengue transmission caused by population size (*14*) and demographics (*15*), the 21 treatment cities were divided into 5 groups by population size: 18,000–21,000, 35,000–60,000, 70,000–90,000, 100,000–140,000 and 150,000–300,000 for comparison with control cities (Figure 2, panel B). Cities within Minas Gerais that did not implement MID were referred to as control cities. There were 147 control cities that could be grouped into a distribution of population sizes of treatment cities.

To compare this large sample size in a case–control format to only 21 MID cities, we generated 1,000 random sets of 21 control cities with the same population distribution as the MID cities. Next, we calculated the relative difference in incidence (RI) for the same period before and after the start of surveillance for each treatment city (i.e., incidence for x time before MID/incidence for x time after MID). Likewise, for each set of the control cities, we calculated RI using the distribution of time frames in each group of treatment cities (Figure 2, panel C) matched to the corresponding group in control cities. Lastly, we calculated the median RI for each of the 1,000 sets of control cities and the set of treatment cities and calculated the difference (*d* = *RI*_control_ – *RI*_MID_). Under the null hypothesis that MID had no effect at decreasing RI, the median of the 1,000 differences would be 0. We tested this hypothesis using a sign test. The alternative hypothesis was that the median of the 1,000 differences would be significantly >0 if MID decreased the RI of treatment cities.

We identified factors that affected the effectiveness and cost-effectiveness of MID by using a generalized linear model (γ distribution, log link) with either RI or US dollars/prevented case as response variables. Factors considered were population size (PS); distance to 3 large populations (D3L); distance to 3 high-incidence populations; a ranking system for the effectiveness of using MID (PED); a measure of average mosquito infestation during the dengue season in 2011 (IMFA); population density; income per capita; and an index between 0 and 1 that included employment, income, education, and health, all with equal weight. Distances were the sum of Euclidian distance to 3 cities with population size (D3L) or density in the 90th percentile. We fit each variable individually and fit all possible linear combinations of the 8 variables.

We chose between competing models by using delta Akaike Information Criterion (ΔAIC = AIC of intercept only model – AIC of target model). A higher ΔAIC indicates a better model of the data. When comparing nested models that differed by only 1 factor, 2 AIC points is considered a significant difference (α = 0.05). Statistics for all single-variable models, model selection results, and fits of the best multivariable and full models are shown in of Technical Appendix 2 Tables 1, 2, and 3.

**Figure 3:**
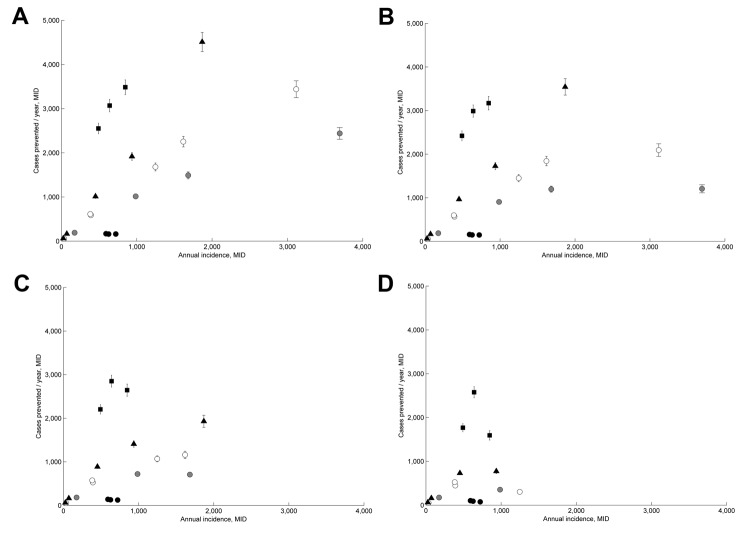
Effectiveness of Monitoramento Inteligente da Dengue (Intelligent Dengue Monitoring System [MID]), Minais Gerais, Brazil, mid-2009–mid 2011. Predicted number of dengue fever cases prevented per year during the time of MID are plotted against the annual incidence of dengue fever in each city during the same time. K is a percentage value of the population size in a city. Error bars indicate 2 SE. A) 29,533 cases were prevented when K = 50%. B) 24,263 cases were prevented when K = 20%. C) 16,578 cases were prevented when K = 10%. D) 9,219 cases were prevented when K = 5%. Shaded symbols distinguish population size classes as follows: black circles indicate 18,000–21,000; gray circles indicate 35,000–60,000; white circles indicate 70,000–90,000; triangles indicate 100,000–140,000; squares indicate 150,000–300,000.

We estimated the number of cases prevented by MID by predicting the number of cases that would have occurred in the absence of MID and taking the difference between those and the number of observed cases (i.e., cases prevented/year = predicted cases in the absence of MID [*E*] – observed annual cases [*O*]). We calculated *E* by using a logistic model according to the equation *E_i_* = *d_i_O_i_*(1 – *O_i_*/*K_i_*), where *K* is the maximum number of possible cases in city *i*. The logic is that the number of cases prevented depends on the estimated growth coefficient (*d = RI_control_ – RI_MID_*) and the observed cases (*O*) but is capped by a theoretical maximum on the number of possible new cases (*K*). In the main text, we assumed that *K* was equal to 30% of the population in city *i,* which has been observed (*16*). However, we also considered higher and lower values of *K* (5%, 10%, 20%, and 50%) (Figure 3).

### Cost Data

All costs were in US dollars. Costs per dengue case were taken from the report of Sheppard et al. (*7*). They calculated direct and indirect costs for ambulatory ($69 and $317, respectively) and hospitalized ($428 and $460, respectively) case-patients. We considered dengue fever case-patients to be ambulatory and dengue hemorrhagic fever case-patients, dengue shock syndrome case-patients, and case-patients who died to be hospitalized. We did not distinguish deaths (0.045% of case-patients) from severe cases (0.38% of case-patients) because we could not obtain the age distribution of deaths and gross domestic product estimates from each city. Indirect costs assumed an average of 4.5 days of lost work for ambulatory case-patients and 14 days for hospitalized case-patients (*7*). Estimates of indirect costs per case were adjusted to account for case-patients who did not miss work by using the age distribution of case-patients in Brazil (*7*).

Total costs for MID in the 21 cities were measured directly by Ecovec SA (Table). MID costs in individual cities varied from $25,566 to $163,944 (Technical Appendix 2 Table 4). Cost-effectiveness per city was calculated as the measured cost of MID in a given city divided by its number of cases prevented, as estimated from the model. In Minas Gerais, vector control activities are conducted according to guidelines of the National Program for Dengue Control (*17*) and the state department of health in Minas Gerais. Government resources are apportioned to cities on the basis of their population size and history of dengue incidence. Thus, we assumed that the per capita cost of control was similar in each treatment city. To estimate the cost of mosquito control activities in each city, we took the per capita cost ($1.11) from a study in Sao Paulo, Brazil, in 2005 (*18*) and multiplied this cost by the population size in each city. The previous study measured 3 components of dengue control costs: vector control activities (larval survey, insecticide spraying); laboratory activities (entomology and serologic analysis); and public education and database maintenance. Labor comprised ≈60% of costs, and materials needed for conducting the work comprised 31% of costs (*18*).

**Table:** Total costs of MID in 21 cities, Brazil, 2007–2011*

Product or service	Cost in US dollars (%)
Royalties to UFMG and FAPEMIG	
MID†	29,918.96 (2.0)
MI-Virus†	38,894.65 (2.6)
Consumables (licensing)	
MosquiTRAP†	77,533.50 (5.2)
Sticky card	112,776.00 (7.5)
AtrAedes†	131,572.00 (8.8)
Web software	21,000.00 (1.4)
Mobile software	96,041.00 (6.4)
Services	
MI-Vírus kit and analyses	58,485.00 (3.9)
MID	61,303.96 (4.1)
Shipping and freight	
MI-Virus and traps	11,056.45 (0.7)
Stationary and materials	668.40 (0.0)
Technical and supervision (employees and taxes)	
Technical support at Ecovec SA, 12 h/d	115,499.00 (7.7)
Technical support at cities visited	80,208.00 (5.4)
Full-time biologist	372,520.54 (24.9)
Technical visits on site	19,200.00 (1.3)
Taxes	269,270.66 (18.0)
Total	1,495,948.13 (100.0)

*MID, Monitoramento Inteligente da Dengue (Intelligent Dengue Monitoring System); UFMG, Universidade Federal de Minas Gerais; FAPEMIG, Fundação de Amparo a Pesquisa do Estado de Minas Gerais; MI-Virus, Intelligent Virus Monitoring System; MosquiTRAP, fixed-position trap designed to capture gravid mosquitoes; AtrAedes, synthetic ovipostion attractant.  †Manufactured by Ecovec SA (Belo Horizonte, Brazil).

For each treatment city, we calculated the direct, indirect, and total costs of dengue. Direct costs comprised medical and nonmedical direct costs, as well as vector control and MID. Indirect costs comprised costs for lost wages and MID costs in treatment cities. Costs for MID cities were calculated from the number of observed cases (divided into ambulatory and hospitalized case-patients). Similarly, the estimated costs of dengue in the absence of MID were divided into ambulatory and hospitalized case-patients by multiplying the sum of the number of observed cases plus the number of prevented cases by the proportion of observed cases in persons who were ambulatory or hospitalized. The dollars saved annually were calculated by subtracting the cost of dengue in MID cities from the predicted cost of dengue if MID were not implemented. Underreporting was not accounted for because we had no city-specific data to inform estimates. Costs were not discounted because we considered all cases to be nonfatal and our study period was only 2.5 years.

## Results

**Figure 4:**
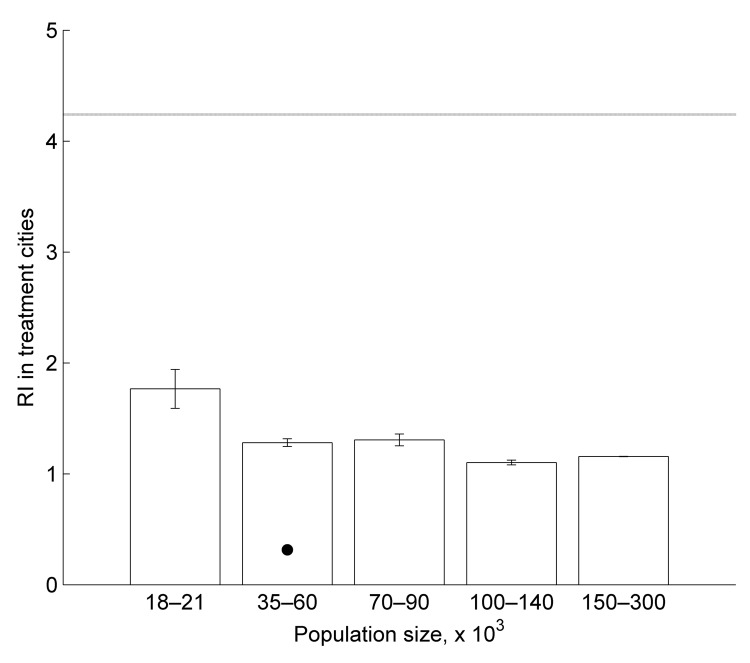
Mean relative difference in incidence (RI) of dengue fever cases for treatment cities grouped by population size using Monitoramento Inteligente da Dengue (Intelligent Dengue Monitoring System), Minas Gerais, Brazil, mid-2009–mid 2011. Horizontal line indicates mean RI for the 1,000 median RI of control city sets. Error bars indicate 2 SD. Error bars for the largest population size group are too small to be shown. The black dot is an outlier that was excluded from the general linear model results.

The annual incidence of dengue in control cities varied more widely than in treatment cities, and the median annual incidence in treatment cities was generally higher (Figure 2, panel A). However, there was a trend of decreased difference in incidence between annual incidence in treatment cities relative to control cities during the years (2010–2011) in which MID was used during the peak dengue season (January–May or June) (Figure 2, panel A). This trend was confirmed by the finding that the RI before and after the time frame of MID was 2.7× higher (a decrease from 4.0 in control cities to 1.3 in treatment cities; 68%), in control cities relative to those that used MID (*z* = −31.59, p<0.0001) (Figure 2, panel D). The RI for treatment cities for each population group is shown in Figure 4.

The most parsimonious generalized linear model of RI in MID cities included PS and IDFM (Technical Appendix 2 Table 2). PS and IDFM showed a negative correlation with RI, although the correlation of PS was marginally not significant (p = 0.083 for PS and p = 0.0023 for IDFM) (Technical Appendix 2 Table 3). This finding indicates that MID effectiveness was higher in cities with stronger economies and that there is a trend of higher effectiveness in larger populations. In contrast, the most parsimonious model of cost-effectiveness included IMFA and D3L (Technical Appendix 2 Table 2). IMFA and D3L showed a negative correlation with cost-effectiveness (p = 0.0086 and p = 0.032, respectively) (Technical Appendix 2 Table 3). Thus, cost-effectiveness was higher in cities with higher mosquito infestation levels and cities that were farther from cities with large populations.

**Figure 5:**
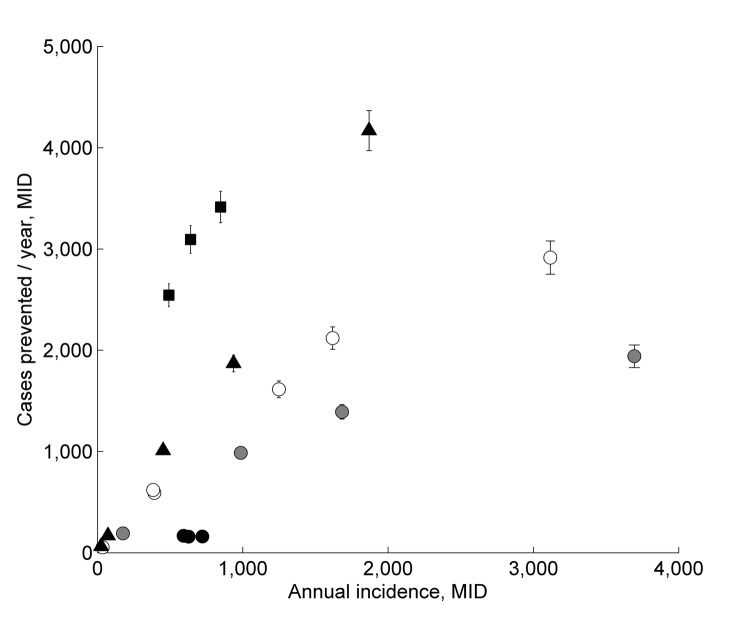
Effectiveness of Monitoramento Inteligente da Dengue (Intelligent Dengue Monitoring System [MID]), Minas Gerais, Brazil, mid-2009–mid-2011. Predicted number of dengue fever cases prevented per year during the time of MID are plotted against the annual incidence of dengue fever cases in each city during the same time. A total of 27,191 cases were prevented. Cases prevented/year = predicted cases in the absence of MID (*E*) – observed annual cases (*O*), where *E_i_* = *d_i_O_i_*(1 – *O_i_*/*K_i_*), *d* is the difference between median relative difference (RI) in incidence in control cities (mean 1,000 datasets) minus the RI in each treatment city, and K is 30% of the population size in city *i*. Error bars indicate 2 SE of the number of predicted cases that were prevented (points without bars are shown because the SEs are smaller than the size of the point). Shaded symbols distinguish population size classes as follows: black circles indicate 18,000–21,000; gray circles indicate 35,000–60,000; white circles indicate 70,000–90,000; triangles indicate 100,000–140,000; squares indicate 150,000–300,000.

**Figure 6:**
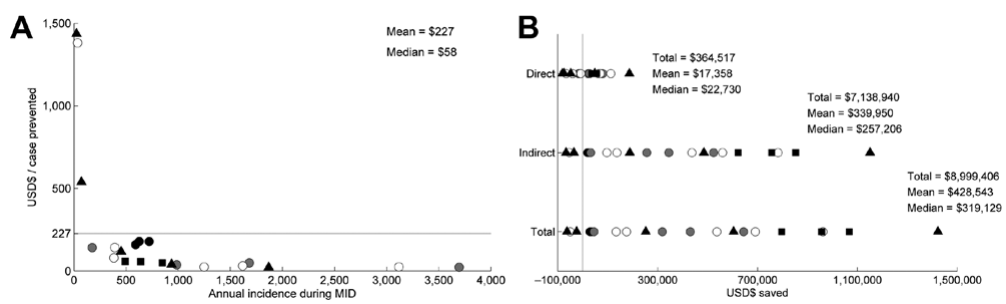
Cost-effectiveness of and savings from Monitoramento Inteligente da Dengue (Intelligent Dengue Monitoring System [MID]), Minas Gerais, Brazil, mid-2009–mid-2011. A) For cost-effectiveness, the number of US dollars (USD$) spent per dengue fever case prevented is plotted against the annual incidence of dengue fever cases during MID for the city. Each point represents cost-effectiveness for a city. Points are coded by population size classes. Horizontal line indicates average cost-effectiveness ($227) per case prevented. B) Savings for each cost component from the benefits of MID. Direct savings include only health care, nonmedical direct savings, and vector control savings. Indirect savings include only savings in the work force. Total savings include direct and indirect savings. Negative values indicate dollars lost because of implementing MID. Vertical line indicates 0. Shaded symbols distinguish population size classes as follows: black circles indicate 18,000–21,000; gray circles indicate 35,000–60,000; white circles indicate 70,000–90,000; triangles indicate 100,000–140,000; squares indicate 150,000–300,000.

Under the assumption that dengue could affect 30% of a population, we estimated that the number of cases prevented by MID annually in the largest cities (>130,000 inhabitants) was 2,300–3,900 (Figure 5). In the smallest cities (<40,000 inhabitants), these estimates decreased to 143–182, and the total number in all 21 cities was 27,191. However, these numbers depend on the assumed number of potentially susceptible persons (Figure 3). The average cost-effectiveness was $227/case prevented, which was driven mainly by a few larger values (Figure 6, panel A). The median value was $58, indicating that the average value was higher than the cost-effectiveness value in most cities. The number of cases prevented translated to net total savings of $8,999,406 annually. Savings in health care and vector control costs was $364,517, and savings in lost wages was $7,138,940 (Figure 6, panel B; Technical Appendix 2 Table 4).

## Discussion

Accurate estimates of dengue incidence and its economic effects are more limited (*16*,*19*) than are estimates of other infectious diseases that pose similarly serious public health threats. This finding is caused mainly by high variability in clinical disease, high underreporting rates, and lack of studies that directly measure the efficacy of controls. Consequently, only a few studies have demonstrated the cost-effectiveness of vector control activities (*19*–*21*). In one of these studies, targeted source reduction was more effective than nontargeted vector control, reducing vector abundance by 52%–82% depending on the country (*21*). Another study found that targeted vector control reduced the dengue case load by 53% (*20*). Our estimate of a 68% reduction in incidence caused by targeted control efforts by MID was higher than that in the study by Suaya et al. (*20*). One reason may be geographic differences in the effects of source reduction methods (the previous study was conducted in Cambodia). Alternatively, our higher estimate may be caused by implementation of MID at a fine spatial scale over a broader area, which produced higher intervention efficacy.

The trend of increased effectiveness in larger populations might not be significant in the multivariable model (which includes IDFM) because PS and IDFM showed a positive correlation (*r* = 0.53). The single-variable model results suggest that MID may be more effective in larger populations (Technical Appendix 2 Table 1). The fact that MID was more cost-effective in cities with higher mosquito infestation levels emphasizes the power of targeting vector control practices to areas in which gravid mosquitoes are most abundant. A possible reason for higher cost-effectiveness in cities that were farther from large cities could be that proximity to larger cities may enable a higher proportion of cases that were contracted elsewhere (i.e., during travel or commuting to large metropolitan areas) (*22*,*23*).

PED, a measure of MID quality, was not correlated with effectiveness or cost-effectiveness of MID. This result suggests that variation in the force of infection between cities overwhelmed differences in PED, the current measure of PED is inaccurate, or both. The relationship between mosquito infestation and human incidence is highly variable in space and time (*24*–*26*). Studies of dengue virus serotype circulation in Brazil have found dominance of a single serotype during any given year, and different genotypes within the serotypic groups have caused large, severe outbreaks because of reduced population immunity (*5*,*27*–*29*). Thus, variations between cities in novel genotype dynamics might have affected variation in RI because of PED. In addition, PED pertains only to MID activities and does not evaluate control activities. It is likely that the quality with which cities conduct prescribed control practices varies, which could also explain the lack of relationship between PED and RI. Furthermore, PED is assessed by a yes or no checklist for MID activities, rather than by an evaluation of the quality of each activity. If only minor activities constitute most of the variation in PED, then little meaningful variation in MID quality between cities might be observed. Regardless, the lack of relationship between PED and efficacy of MID suggests that an additional method for assessing MID quality, perhaps through collaboration with city control personnel, might be useful for maintaining, standardizing, and improving quality.

One caveat to our method of estimating cost-effectiveness is that it was necessary to estimate the number of susceptible hosts in the absence of MID (we used 30%) to predict the number of cases that would be prevented. Although 30% is not unreasonable based on previous studies (*16*), the maximum incidence observed in a given city in Minas Gerais during 2007–2010 was only 8.8%. This discrepancy was partly caused by underreporting, which was not accounted for in our study. Nevertheless, we also provided predictions for lower values of *K* to understand how it could affect our estimates. When fewer hosts are susceptible, the number of cases prevented is also lower, which decreases the cost-effectiveness of MID. Thus, previous large outbreaks with the same serotype and vaccination programs would be expected to decrease the cost-effectiveness of MID. Another caveat to our assumption is that *K* varies by city because of historical disease patterns and other factors. Collection of longitudinal serologic data would be useful for more accurate, city-specific predictions of *K*. Last, our study used previously estimated costs for control activities, health care, and lost wages. These costs were per capita estimates that we assumed could be extrapolated to each city equally. The accuracy of our results could be improved through microcosting analyses within each city.

Although MID showed an average cost-effectiveness value of $227 (median $58) per case prevented in Minas Gerais, the average value increased to $616 in 6 moderately sized cities (population 73,000–117,000) that did not show any savings in direct costs (Technical Appendix 2 Table 4). Three of the cities saved on indirect costs and total costs, but the 3 other cities (Joao Monlevade, Itabira, and Conselheiro Lafeite) had a net loss of up to $81,042 in direct costs and $66,246 in indirect costs because of incorporating MID into their budgets. These 3 cities had relatively low annual dengue incidence in the 2 years before MID implementation (12, 18, and 72 cases per 100,000 population relative to a range of 104–2,014 cases in the other 18 cities except for Paracatu, which had 4 cases). Thus, in general, cities with annual incidences of >72 cases per year were more likely to have higher MID cost-effectiveness.

Furthermore, cities in which MID was implemented had historically high dengue incidences relative to control cities (mean ± SD 2007 and 2008 were 549.1 ± 592 in MID cities and 240.4 ± 567.6 in control cities). Thus, average estimates of cost-effectiveness may be high in cities in which MID was implemented. However, factors determining incidence patterns in a given city, such as population immunity, infrastructure, or human behavior, may not be static over time because high population immunity is not protective against novel serotypes (or genotypes with high forces of infection) and human behavior and infrastructure are continually changing. A predictive model of serotype dynamics across cities formulated on the basis of serologic data would be useful for decisions on which cities should implement MID so that the most cost-effective strategy can be achieved statewide.

Our study showed that MID is generally effective for decreasing case loads and suggested that an MID strategy is theoretically better than other strategies. Although MID cost-effectiveness varied between cities, implementation of MID saved hundreds of thousands of dollars on health care and ≈7 million dollars in lost wages statewide, and half the cities had cost-effectiveness values <$58. Furthermore, these numbers are underestimates because our study did not account for underreporting or additional costs from deaths. Investing more effort into integrating MID strategies and costs with vector control operations, and standardizing the MID-based control system across cities, should help to increase MID cost-effectiveness.

Technical Appendix 1 Monitoramento Inteligente da Dengue (Intelligent Dengue Monitoring System), Minas Gerais, Brazil.

Technical Appendix 2 Monitoramento Inteligente da Dengue (Intelligent Dengue Monitoring System), Minas Gerais, Brazil. Table 1, Factors that affected effectiveness and cost-effectiveness; Table 2, Top 5 multivariate models; Table 3, Fit of multivariable models; Table 4, Cost-effectiveness and savings for each city.
