# Supplementary material for: Cost-effectiveness of Novel System of Mosquito Surveillance and Control, Brazil
Source: Emerg Infect Dis. 2013 Apr;19(4):542–50. doi: 10.3201/eid1904.120117 (PMC3647717; doi:10.3201/eid1904.120117)
Supplement: Technical Appendix 1 — Monitoramento Inteligente da Dengue (Intelligent Dengue Monitoring System), Minas Gerais, Brazil. [file 12-0117-Techapp-s1.pdf]

Article DOI: http://dx.doi.org/10.3201/eid1904.120117

Cost-effectiveness of Novel System of Mosquito Surveillance and Control, Brazil

Technical Appendix 1

Monitoramento Inteligente da Dengue (Intelligent Dengue Monitoring System), Minas Gerais, Brazil

Monitoramento Inteligente da Dengue (Intelligent Dengue Monitoring System [MID]) is a patented technology service developed by a partnership of the Universidade Federal de Minas Gerais and the spin-off company Ecovec SA (Belo Horizonte, Brazil). The MID consists of MosquiTRAP, a sticky trap that targets gravid, adult female *Aedes aegypti* mosquitoes by using a synthetic oviposition attractant (Atr*Aedes*); mobile phones with MID mobile software, which sends trap data to the Ecovec SA database instantaneously from the trap location on a weekly basis; and an MID Web-based system, which automatically updates the field data into current infestation levels. Because traps are placed ≈300 m apart, following a 300 m × 300 m grid design, the mean number of female *Ae. aegypti* mosquitoes by city, neighborhood, and block are available for control personnel to target hotspots in highly infested neighborhoods. MID differs from traditional vector surveillance methods by specifically targeting female adult mosquitoes, identifying dengue vectors during trap inspection, reducing labor intensity, and providing higher resolution geospatial trap data.

MID activities are designed to be standardized across cities. Quality control is monitored monthly through a performance evaluation implemented on the Web-based system. An index of MID quality, known as the Dengue Excellence Program (PED) is calculated as the percentage of activities completed. PED can be used for assessing efficacy of MID given effort and for facilitating improvement of activities. The PED program standardizes only MID activities (which excludes control activities because they are conducted according to individual city programs and personnel) and consists of 8 standards: 1) inspection of traps, 2) mosquito capture for virologic analyses, 3) field supervision of field workers, 4) materials and their management, 5) trap replacement, 6) team management, 7) dengue control intensification on the basis of MID data,

and 8) general supervision and management of all activities (only for the MID general supervisor).

Dengue control is not conducted by Ecovec SA or the MID team. Rather, each city employs control personnel and decides how to allot control resources on the basis of guidelines set by the Brazilian Health Ministry (Dengue Control National Program) (*1*). The biologists at Ecovec SA work with control personnel to help them understand how to use MID results for control purposes. Essentially, this procedure involves prioritizing controls in neighborhoods or individual city blocks in the order of highest current infestation levels (action plan). However, there is variation between cities in the emphasis on MID data for control because some cities combine mosquito infestation data with human incidence data for site prioritization, a choice that is outside the authority of Ecovec SA. Individual cities also choose the frequency at which different control methods are implemented on the basis of their resources, although all types of controls recommended by the Dengue Control National Program are implemented to some degree in all cities. These methods include removal or larvicide treatment of possible breeding sites in and outside houses, adulticide treatment of blocks and neighborhoods, public education, and garbage removal.

Reference

1. Ministry of Health, Brazil. National guidelines for the prevention and control of dengue outbreaks [in Portuguese]. Brasilia (Brazil): The Ministry; 2009.
